# Detection of Potential TNA and RNA Nucleoside Precursors in a Prebiotic Mixture by Pure Shift Diffusion-Ordered NMR Spectroscopy

**DOI:** 10.1002/chem.201202649

**Published:** 2013-02-01

**Authors:** Saidul Islam, Juan A Aguilar, Matthew W Powner, Mathias Nilsson, Gareth A Morris, John D Sutherland

**Affiliations:** [b] School of Chemistry, University of ManchesterOxford Road, Manchester, M13 9PL (UK) E-mail: juan.aguilarmalavia@manchester.ac.uk; [c] Department of Chemistry, University College London, Christopher Ingold Laboratories20 Gordon Street, London, WC1H 0AJ (UK); [d] University of Copenhagen, Faculty of Science, Dept. of Food Science, Quality & TechnologyRolighedsvej 30, 1958 Frederiksberg C (DK); [e] MRC Laboratory of Molecular BiologyHills Road, Cambridge, CB2 0QH (UK)

**Keywords:** diffusion, NMR spectroscopy, prebiotic, RNA, TNA

## Abstract

In the context of prebiotic chemistry, one of the characteristics of mixed nitrogenous-oxygenous chemistry is its propensity to give rise to highly complex reaction mixtures. There is therefore an urgent need to develop improved spectroscopic techniques if onerous chromatographic separations are to be avoided. One potential avenue is the combination of pure shift methodology, in which NMR spectra are measured with greatly improved resolution by suppressing multiplet structure, with diffusion-ordered spectroscopy, in which NMR signals from different species are distinguished through their different rates of diffusion. Such a combination has the added advantage of working with intact mixtures, allowing analyses to be carried out without perturbing mixtures in which chemical entities are part of a network of reactions in equilibrium. As part of a systems chemistry approach towards investigating the self-assembly of potentially prebiotic small molecules, we have analysed the complex mixture arising from mixing glycolaldehyde and cyanamide, in a first application of pure shift DOSY NMR to the characterisation of a partially unknown reaction composition. The work presented illustrates the potential of pure shift DOSY to be applied to chemistries that give rise to mixtures of compounds in which the NMR signal resolution is poor. The direct formation of potential RNA and TNA nucleoside precursors, amongst other adducts, was observed. These preliminary observations may have implications for the potentially prebiotic assembly chemistry of pyrimidine threonucleotides, and therefore of TNA, by using recently reported chemistries that yield the activated pyridimidine ribonucleotides.

## Introduction

At some point in the origin of life, an informational polymer is believed to have self-assembled by a predisposed chemical route. The “RNA world” hypothesis states that RNA may have been the sole propagator of genetic information and heritable catalytic function in life on the primitive earth.[1–3] The inherent selectivity issues of an abiotic synthesis of RNA[4, 5] have led some to postulate that it may have been predated by “simpler” nucleic acids.[6, 7] This “simpler” genetic polymer is assumed to have self-assembled more readily, and biology based on this (bio)polymer aided the transition to life based on RNA following what is described as a “genetic takeover”.[8]

**Figure 1:**
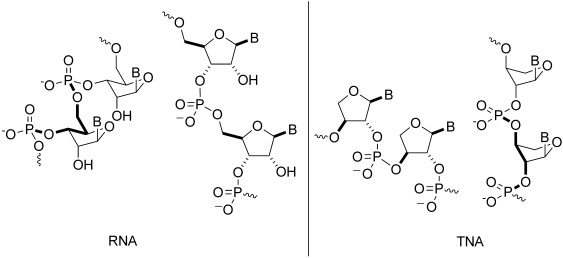
Idealised pairing conformations, constitutions and configurations of RNA and TNA.

The seminal contributions of Eschenmoser and his co-workers on the chemical etiology of the nucleic acid structure led to the discovery of various informational base-pairing systems that are constitutionally related to RNA.[9–11] Threose nucleic acid (TNA), an oligonucleotide consisting of a four-carbon threofuranosyl sugar, a five covalent bond *trans*-diaxial phosphodiester backbone, and only three stereogenic centres, has attracted particular interest (Figure 1).[12] The structural simplifications of TNA (relative to RNA), and its ability to Watson–Crick base-pair not only to complementary strands of TNA, but also to RNA and DNA, have led to the suggestion that TNA may have predated RNA in the origin of life.[13]

Various studies involving TNA have provided support for this theory. For example, it was demonstrated that some DNA polymerases can synthesise short sequences of DNA on a TNA template.[14] Subsequently, it was shown that various polymerases could produce short sequences of TNA on a complementary DNA template.[15, 16] Building on the earlier work of Szostak and co-workers,[17–19] Chaput and co-workers achieved the isolation of a TNA aptamer that can bind to human thrombin with high affinity and specificity.[20] It therefore raises the interesting possibility of isolating TNA enzymes using in vitro selection. More recently, a study led by Holliger demonstrated that genetic information can be stored in and recovered from six alternative genetic polymers, one of which is TNA.[21] Such a process had previously been assumed to occur only between DNA and RNA.

Despite an assumed generational simplicity, primarily as a consequence of the outward appearance of the three-dimensional structure of TNA, there have been no prebiotically plausible syntheses of TNA reported so far. Some authors have argued against the stark and somewhat premature conclusion that RNA was predated by a “simpler” genetic polymer, and statements on the ancestry of RNA should be deferred until a comprehensive investigation of the assembly chemistry of plausible prebiotic small molecules has been completed.[22]

**Scheme 1:**
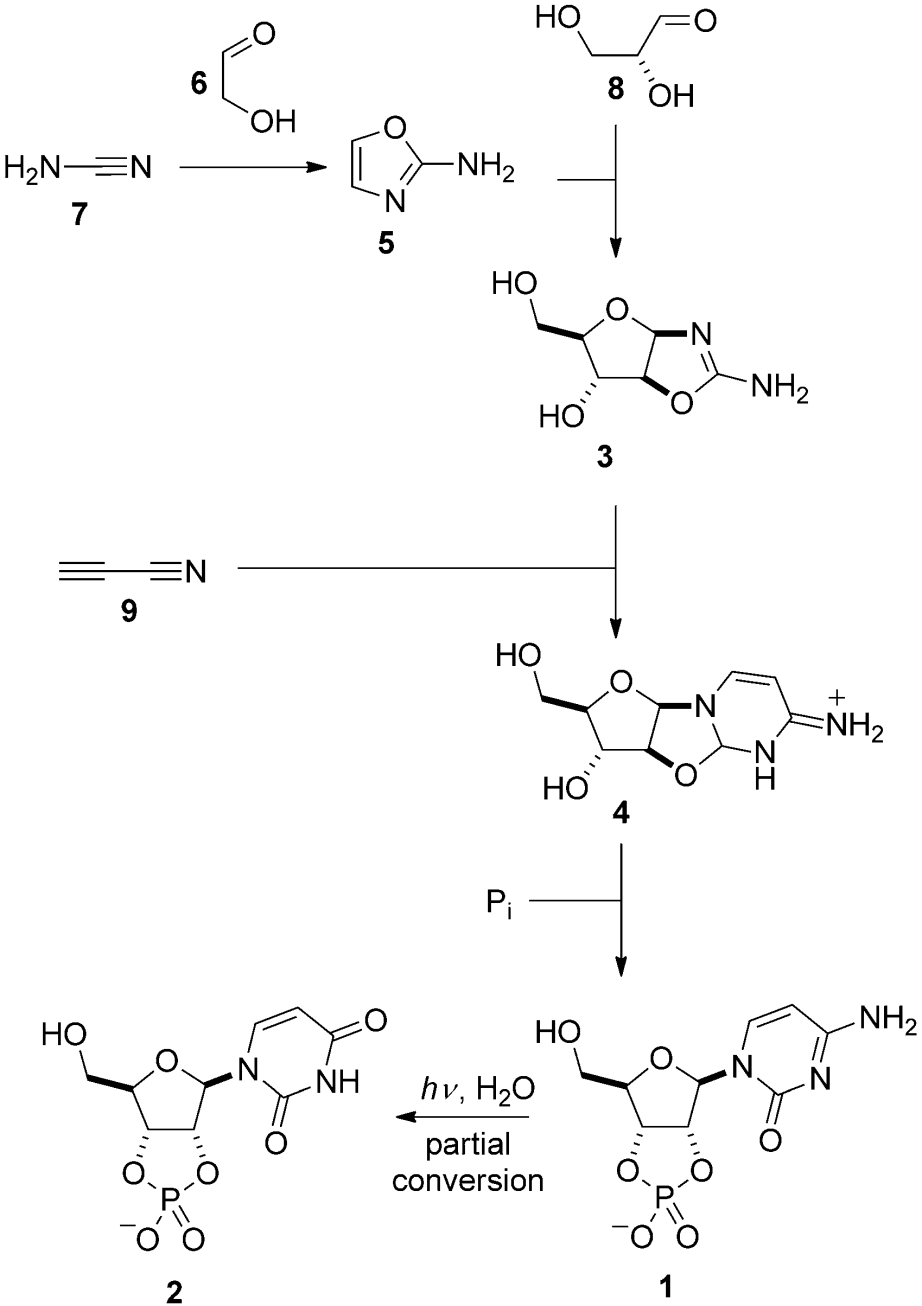
Synthesis of activated pyrimidine ribonucleotides **1** and **2**.

The “RNA world” hypothesis has recently gained major support with a potentially prebiotic synthesis of the activated pyrimidine ribonucleotides **1** and **2**.[23, 24] Both canonical pyrimidine nucleotides are formed in a short sequence of inorganic phosphate mediated steps that bypasses free ribose[25, 26] and preformed nucleobases, and instead proceeds through arabinose aminooxazoline **3** and its corresponding anhydrocytidine intermediate **4** (Scheme 1).[23] Additionally, since the originally reported synthesis and photochemical purification of a racemic mixture of **1** and **2**, the problem of RNA homochirality has now been partially addressed.[27–29]

**Scheme 2:**
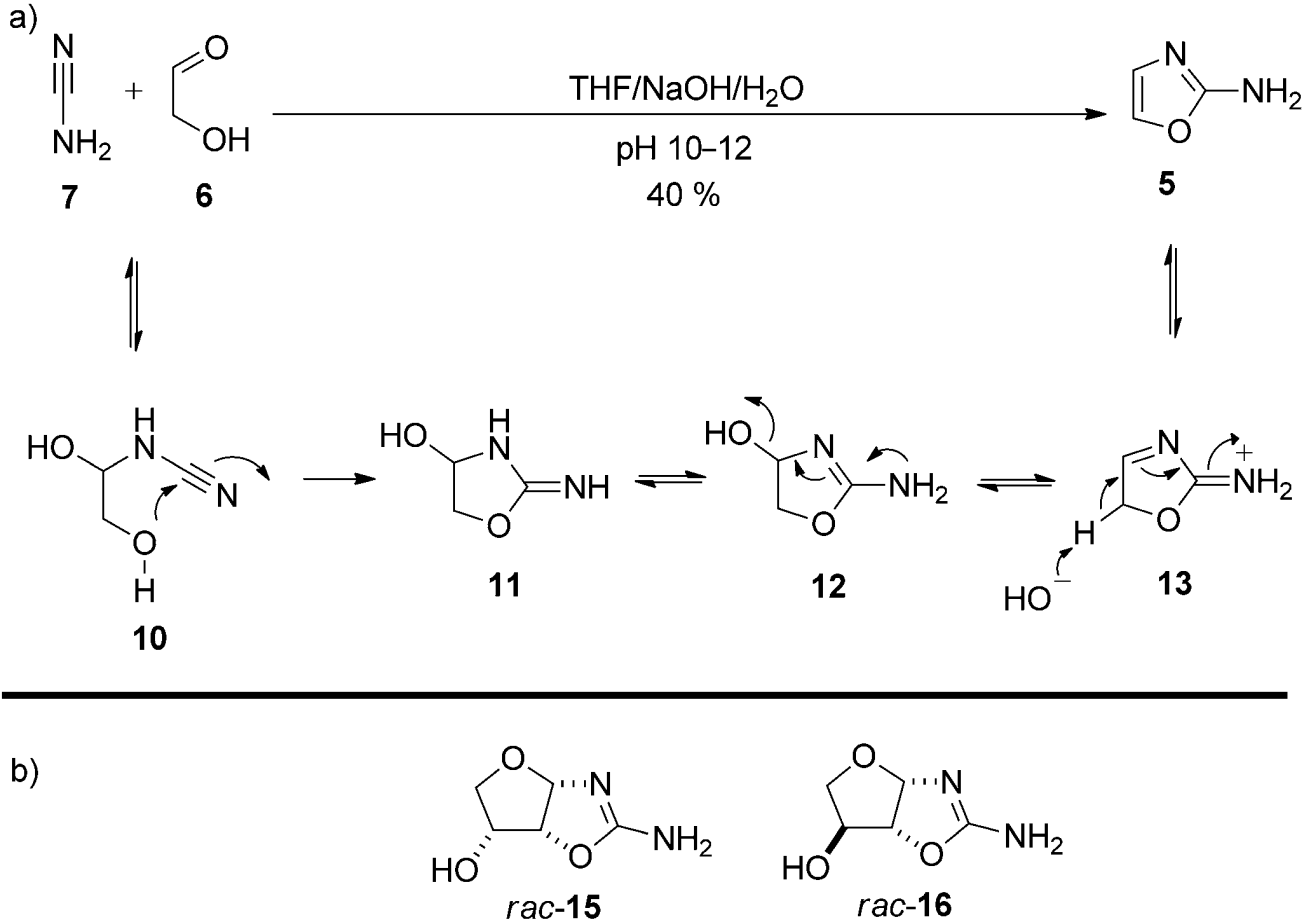
a) Formation of 2-aminooxazole **5** under basic conditions, and b) structures of tetrose aminooxazolines *rac*-**15** and *rac*-**16**.

There are still a number of fundamental problems precluding the acceptance of the “RNA world” hypothesis. Notably, a robust prebiotic synthesis of activated purine β-ribonucleotides, and then the oligomerisation of activated β-ribonucleotides to yield pure [5′→3′] phosphodiester linkages needs to be demonstrated. During our exploration of the assembly chemistry of nucleoside precursors,[27] it was deemed important to investigate the prebiotic synthesis of key nucleotide synthon 2-aminooxazole **5**. Cockerill et al. reported the condensation of glycolaldehyde **6** and cyanamide **7** to yield **5** in aqueous THF at high pH (10–12), as exemplified in Scheme 2 a.[30] However, the instability of glyceraldehyde **8** in alkali renders these conditions incompatible with the assembly of **3** (Scheme 1). Furthermore, there is no evidence to suggest that THF is a potentially prebiotic solvent. The behaviour of **6** and **7** was explored in the absence of organic co-solvents and closer to neutral pH and temperature. Eventually, it was shown that a stoichiometric mixture of **6** and **7**, buffered at pH 7 with inorganic phosphate, improved the yield of 2-aminooxazole **5** to more than 80 %.[23]

**Figure 2:**
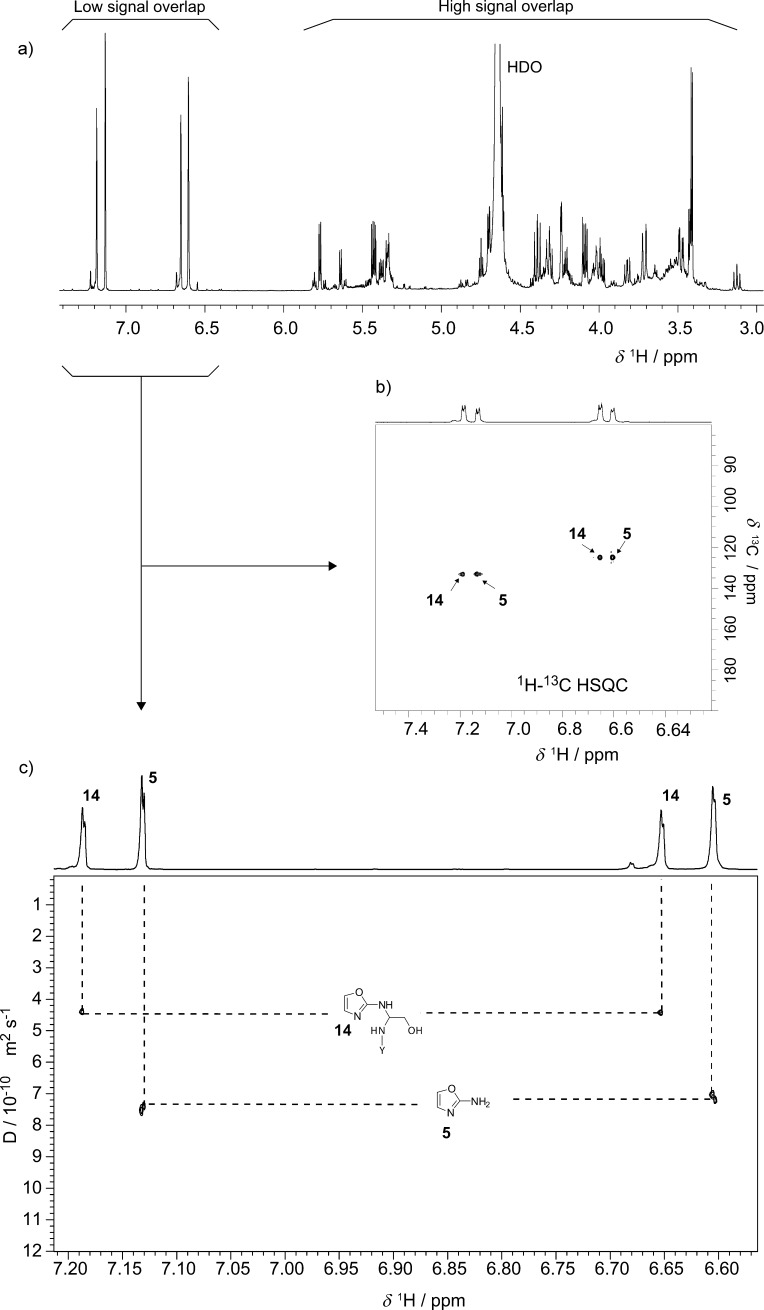
a) ^1^H, and b) ^1^H-^13^C HSQC spectra showing the ^1^H chemical shift region (*δ*=6.5–7.5 ppm) in which the signals corresponding to 2-aminooxazoline **5** are expected to be seen. Spectrum (b) shows only two chemical shifts for carbon. A ^1^H-^15^N HMBC spectrum of the same ^1^H chemical shift range showed all four peaks with the same ^15^N chemical shift. The DOSY spectrum (c), however, shows the presence of two different chemical species; that with the greater diffusion coefficient was assigned to compound **5**, the other to **14**. See the Supporting Information for the full standard DOSY spectrum.

When the reaction was carried out in the absence of inorganic phosphate and incubated at an initial pD of 7 at 40 °C, a complex product distribution was observed by ^1^H NMR spectroscopy (Figure 2 a). To obtain a more complete picture of the chemistry taking place in this mixed oxygenous/nitrogenous system, we attempted to characterise this complex mixture. Because of the outwardly complex nature of the composition, we also considered this mixture to be an ideal candidate for analysis by new pure shift NMR methods,[31–33] alongside an array of standard NMR techniques (^1^H-^1^H COSY, ^1^H-^13^C HSQC, ^1^H-^13^C HMBC and ^1^H DOSY).[34a–f] This is the first time that pure shift diffusion-ordered spectroscopy (DOSY) NMR methodology has been applied to a complex reaction system of partially unknown composition. By using these techniques, we confirmed the direct formation of both TNA nucleoside precursors *rac*-**15** and *rac*-**16** (Scheme 2 b) amongst the products from the reaction of **6** and **7**. We believe these preliminary results may have direct implications for the potentially prebiotic assembly chemistry of pyrimidine threonucleotides and TNA.

## Results and Discussion

**Scheme 3:**
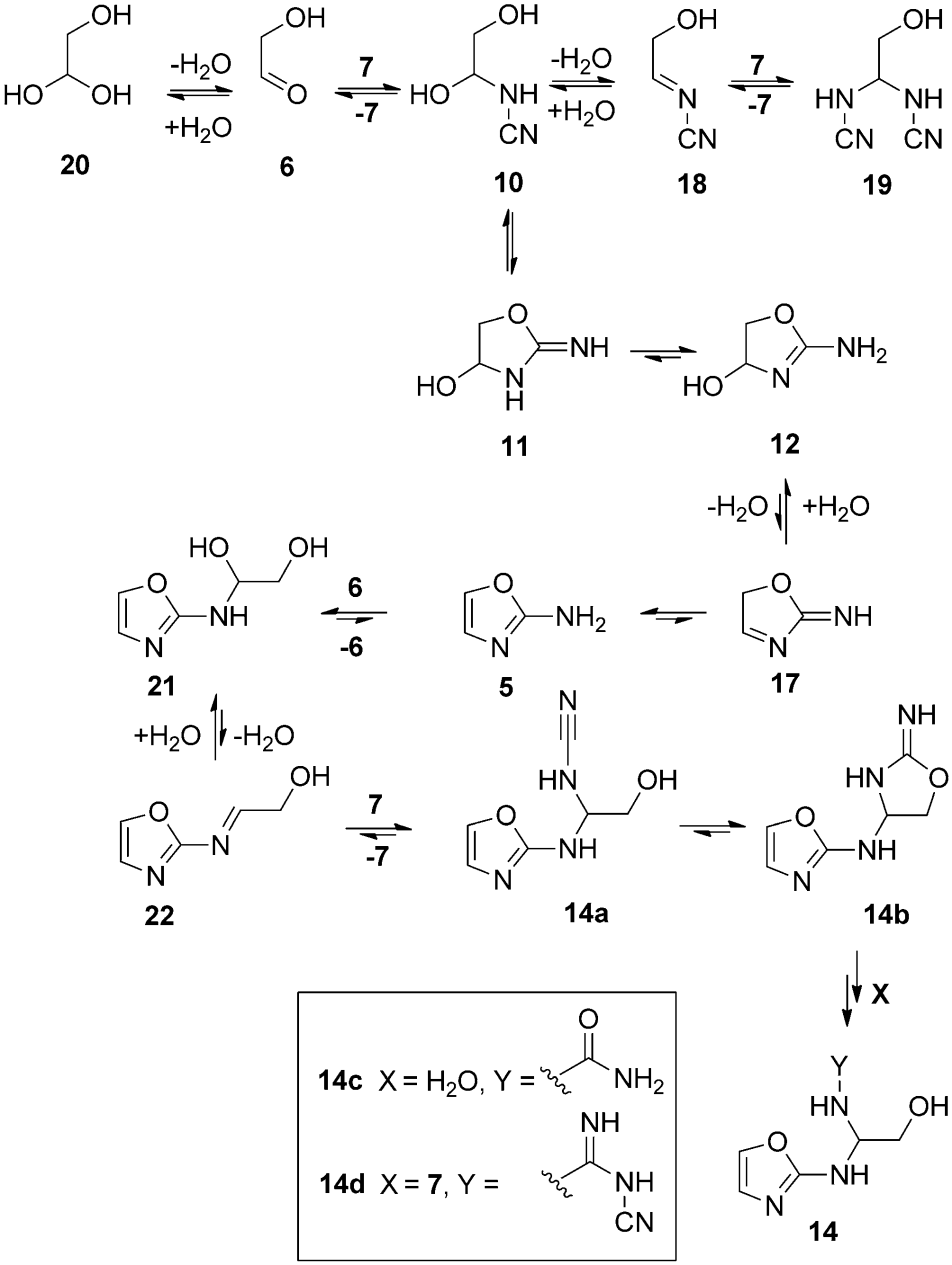
Steps towards the formation of 2-aminooxazole **5**.

One of the characteristics of prebiotic chemistry is its propensity to give rise to complex mixtures. With the emergence of systems chemistry,[24, 37, 38] which concerns itself with the study of molecular entities in mixed chemical systems, the development of improved analytical techniques has become increasingly important. Our group has generally used NMR spectroscopy and spiking methods with synthetically prepared compounds to aid with structural characterisation of potentially prebiotic reaction mixtures. One of the disadvantages of the spiking method is the associated monetary cost and the time required to prepare synthetic standards, and alternatives to such methods are desirable. NMR is one of the most powerful tools available for structure elucidation, although most research in NMR methodology is geared towards analysis of pure compounds, in which identification of spin systems is usually enough. This bias is unfortunate when dealing with mixtures, as there are very few experiments that facilitate the process of identifying the different isolated spin systems that belong to a particular chemical species. This problem is at its most acute when signals overlap, a common occurrence in the spectra of mixtures. Multidimensional experiments have been used both to palliate the problem and to provide structural information; however these experiments are themselves prone to signal overlap, leading to cross-peaks that appear to imply chimeric structures formed by conjoined spin systems that actually belong to different chemical species. In the problem at hand, only the small ^1^H spectral area between *δ*=6.5 and 7.5 ppm appears to be largely free of signal overlap, as evidenced by the proton and ^1^H-^13^C HSQC spectra of Figure 2 a and b. However, the spectroscopic data remain ambiguous because it is not clear whether the two spin systems identified as **5** and a closely related compound with the general structure **14** belong to the same, or to two different, species. The DOSY spectrum of Figure 2 c, however, shows clearly that the diffusion coefficients for systems **5** and **14** are different, and that they therefore correspond to different chemical species of different size. The component with the larger diffusion coefficient was tentatively assigned to **5**, whereas the more-slowly diffusing spin system was attributed to **14**. It must be stressed that absolute and unambiguous assignment of **14** was not possible in the present study. In considering the potential chemistries that could have taken place, possibilities for the potential pendent group Y of **14** are tentatively given as **14 a**, **14 c** or **14 d**, although ^13^C NMR data suggests the **14 c** or **14 d** are likelier than **14 a** (Scheme 3; see the Supporting Information). Ideally the assignment of **14** could be supported by observation of methylene and methine signals with the same diffusion coefficient. Unfortunately, the high degree of overlap in the remainder of the spectrum renders this impossible, because the fitting procedure normally used to determine diffusion coefficients from signal attenuation measurements can only produce accurate results when either there is no overlap between signals, or any signals that overlap have both good signal-to-noise ratio and very different diffusion coefficients.[39] Where two or more signals of similar diffusion coefficient overlap, exponential fitting generally results in a single apparent diffusion coefficient intermediate between the true values. More sophisticated methods such as multivariate analysis of whole experimental datasets[39, 40] can retrieve correct diffusion coefficients for mixtures containing small numbers of species, but fail completely for complex systems such as that studied here, in which a large number of different reaction products give signals in the crowded *δ*=3.0–6.0 ppm region.

**Figure 3:**
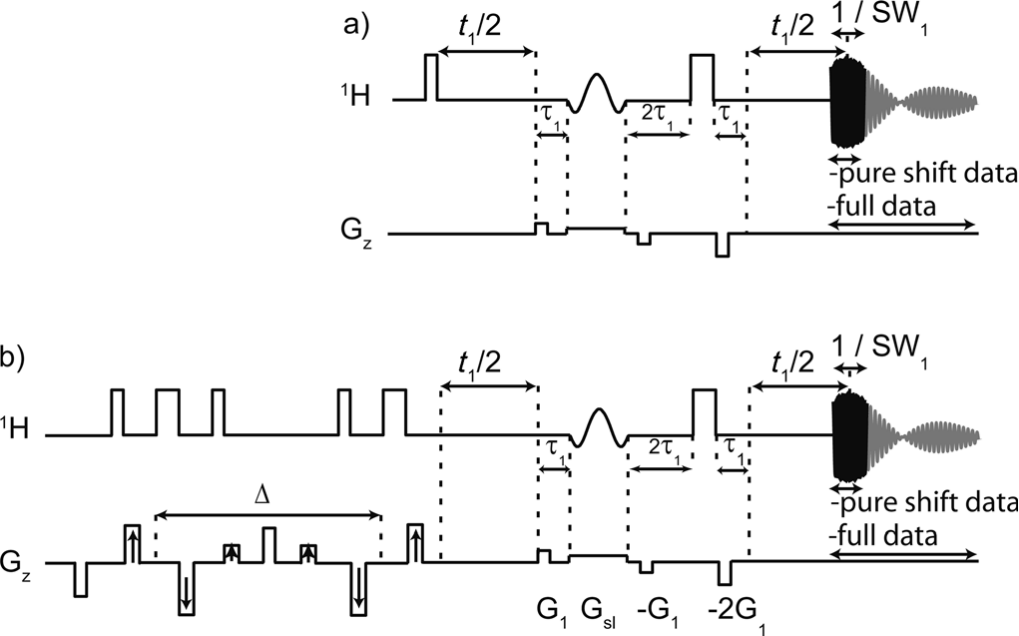
a) Zangger–Sterk pure shift ^1^H, and b) pure shift ^1^H DOSY pulse sequences used in the present work. The dark part of the free induction decay represents the component taken from each increment and assembled into a composite interferogram to produce pure shift data, and the grey additional component that was acquired to allow double Fourier transformation for the purposes of multiplicity determination (a previously unreported extension to the experiment). See the main text for details.

The main obstacle to NMR analysis of this type of reaction mixture is lack of resolving power, so it represents an ideal test for a new class of experiment that collapses homonuclear multiplet structure to give a single spectral line per chemical site, and has hence been termed “pure shift” NMR.[31, 33, 41] One of the most effective of these methods for broadband homonuclear decoupling is based on the work of Zangger and Sterk[42] and has been incorporated into a variety of uni- and multidimensional experiments such as DOSY and TOCSY (Figure 3 b).[31, 32, 43] Reported applications of such methods have hitherto been limited to simple samples containing no more than a few chemical species of comparable concentration. Here, Zangger–Sterk methods are applied to a real and highly complex problem in mixture analysis.

**Figure 4:**
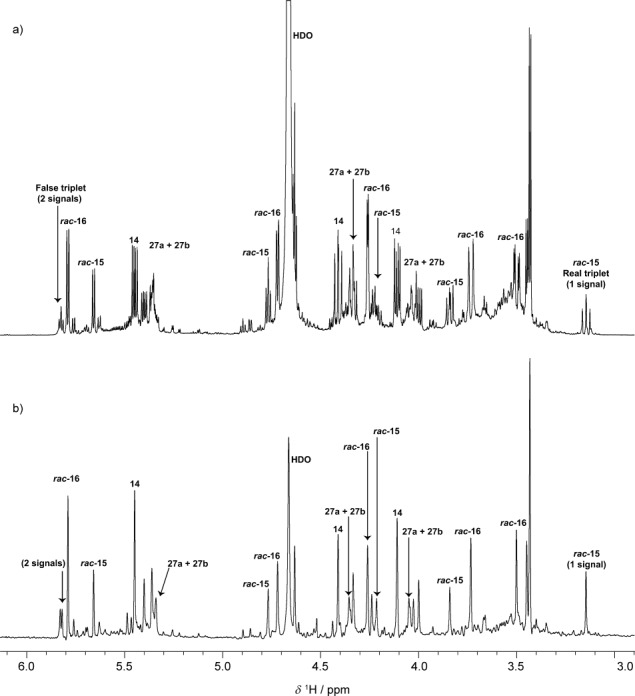
a) ^1^H, and b) pure shift ^1^H NMR spectra of the highly overlapped region, which include signals of the potential TNA precursors tetrose aminooxazolines *rac*-**15** and *rac*-**16** amongst various other adducts.

Exploration of the overlap-rich region *δ*=3.0 to 6.0 ppm (Figure 4) by using the sequence of Figure 3 a greatly simplifies analysis of the ^1^H NMR spectrum, showing for example that the apparent triplet at *δ*=5.81 ppm is actually composed of two chemically shifted doublets, whereas that at *δ*=3.13 ppm is a true triplet. The former signals have not been characterised yet, but the latter was assigned to a potential TNA nucleoside precursor *rac*-**15** (Figure 4).

**Figure 5:**
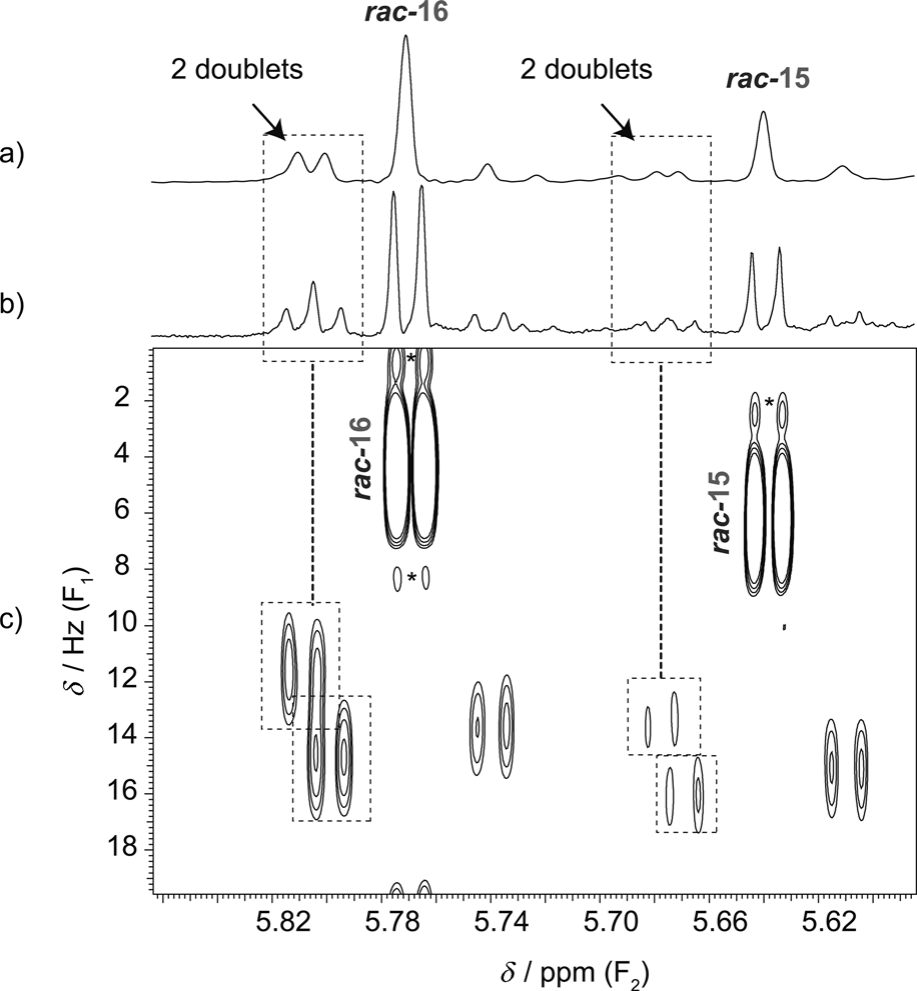
a) 1D pure shift ^1^H NMR, b) normal ^1^H NMR, and c) 2D pure shift NMR multiplicity analysis spectra. In (c), double Fourier transformation of the raw pure shift data that produced spectrum (a) yields a 2D display in which signals are dispersed as normal according to chemical shift and scalar coupling in *F*_2_, but according to shift only in *F*_1_. Sinc wiggles are marked with an asterisk. This 2D processing of the pure shift data provides a means of determining the multiplicities of signals observed in the pure shift 1D NMR spectrum (a) at no extra cost in experiment time. As can be seen in (b), some signals that appear as triplets in the conventional spectrum actually consist of overlapping doublets. The 2D display can also be used to identify artefacts and, where the shift difference between coupled spins is not greater than the bandwidth of the selective pulse used, in cases of incomplete decoupling.

The presence of an array of compounds with different concentrations, and hence different signal amplitudes, places a premium on spectral purity. Zangger–Sterk pure shift methods typically produce small (a few %) artefact signals, usually in the form of weak sidebands at multiples of the *F*_1_ spectral width SW1 (i.e., the inverse of the increment in *t*_1_) used. Normally these sidebands are not seen as they tend to appear at or below the noise level, and when they are seen they rarely represent a problem as they show the same diffusion as their parent signals in DOSY experiments. However, from the point of view of structural characterisation, they are an inconvenience when seen in samples with large differences in concentration, as they may be mistaken for real signals of low-concentration mixture components. Fortunately, the Zangger–Sterk experiment provides a previously unreported means to validate pure shift signals. Although only the black FID component of Figure 3 is needed for the generation of a 1D pure shift spectrum, complete FIDs can be acquired for all *t*_1_ increments so that an absolute value 2D spectrum can be produced by conventional double Fourier transformation (Figure 3). Such a 2D spectrum shows normal chemical shifts and multiplet structure in *F*_2_, but only chemical shifts in *F*_1_; it contains similar information to a 2D*J* (*J*-resolved) spectrum, allowing the multiplet structure due to scalar coupling to be determined for each chemical shift, but is obtained as a by-product of the 1D pure shift experiment at no extra cost in experiment duration or complexity. An example of this is shown in Figure 5. One advantage of complementing the 1D pure shift spectrum with the 2D spectrum is that no signals corresponding to the weak sidebands in the 1D spectrum appear in the 2D, allowing real pure shift signals to be validated if needed by simple comparison. This in turn greatly increases the usable dynamic range of the pure shift experiment, allowing signals from dilute species to be identified with confidence. An alternative 2D method that correlates pure shift and normal spectra is that of Giraud et al.,[44] which is less sensitive but has the significant advantage of allowing phase sensitive display, and has been applied successfully to the analysis of spectra of samples in aligned media.

**Figure 6:**
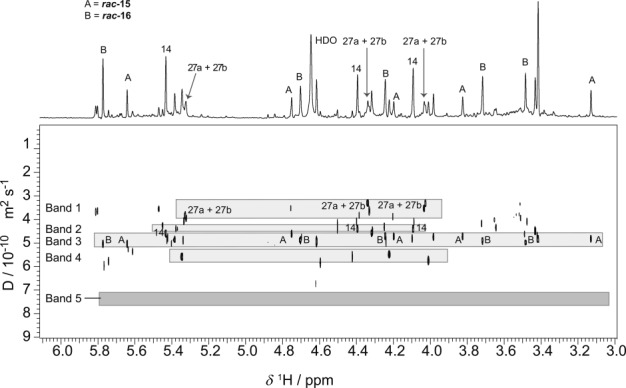
^1^H pure shift Oneshot DOSY spectrum obtained with the pulse sequence of Figure 3 b; shaded rectangles are used to highlight particular diffusion ranges. The corresponding spectrum for the aromatic region (*δ*=6.5–7.5 ppm) is shown in Figure 2. The lack of signals in Band 5 supports the assignment of **5** in Figure 2, whereas the aliphatic signals of **14** are seen in Band 2. Diastereoisomeric tetrose aminooxazolines *rac*-**15** and *rac*-**16** share the same diffusion band as expected. The structures and assignments shown are based on the combined evidence of Figure 2, Figure 4, Figure 5 and of the conventional 2D-NMR experiments (see the Supporting Information).

**Scheme 4:**
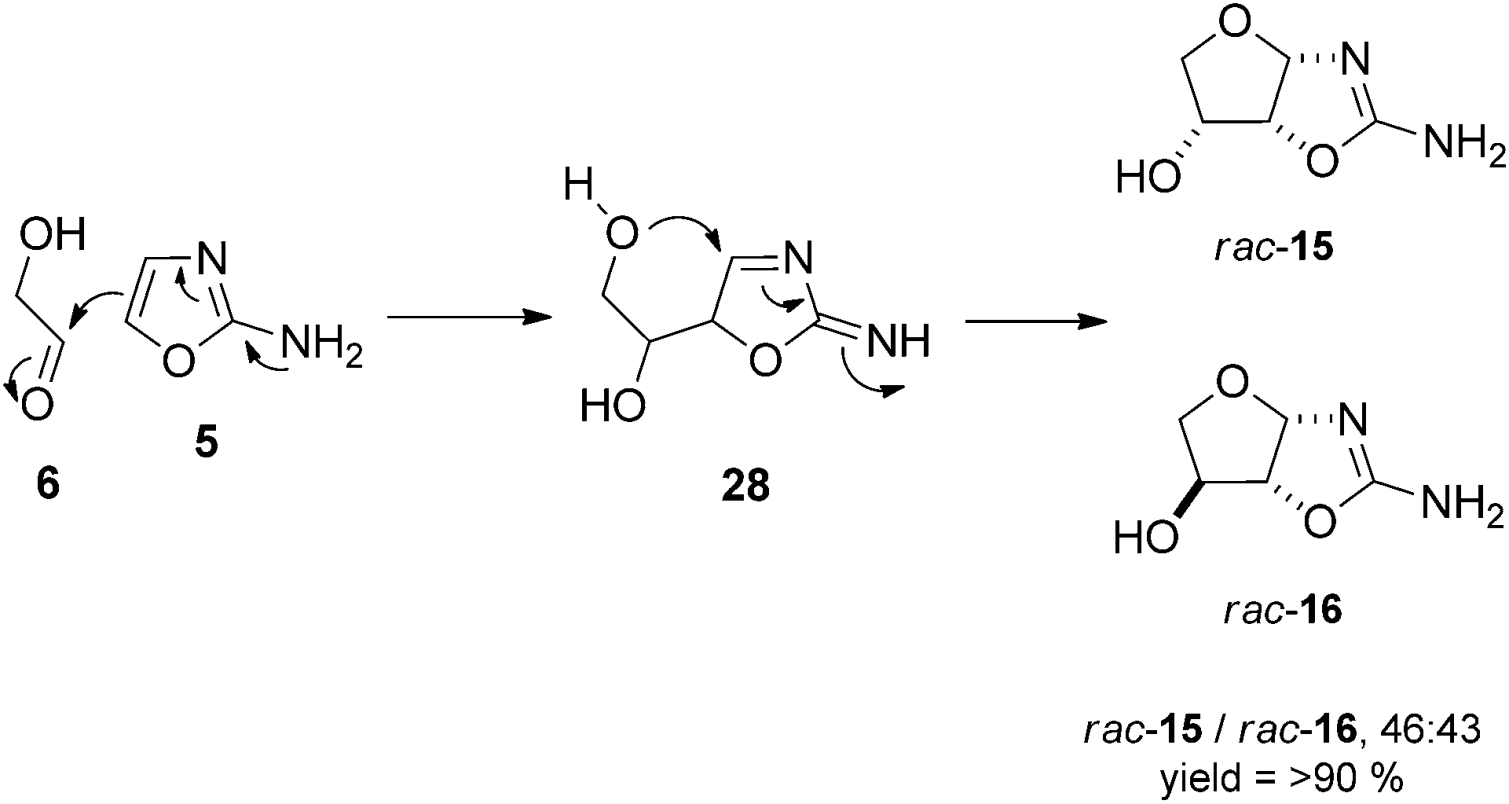
The reaction of isolated 2-aminooxazole **5** with glycolaldehyde **6** under neutral unbuffered conditions in water.

**Figure 7:**
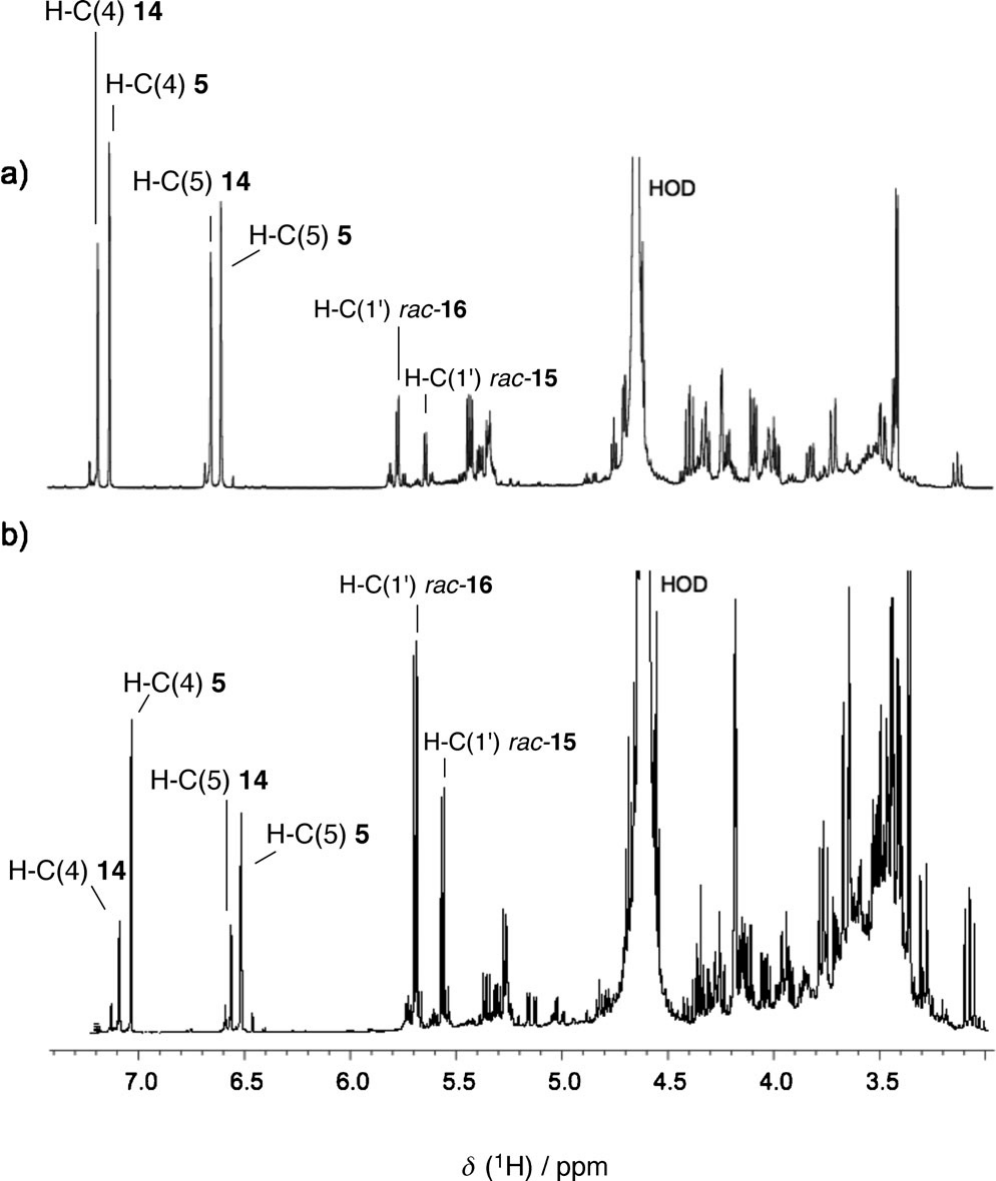
^1^H NMR spectra (400 MHz, D_2_O) comparison of the unbuffered reactions of glycolaldehyde **6** and cyanamide **7** in a: a) 1:1, and b) 2:1 mixture. Greater signal complexity between the *δ*=3.4–3.8 ppm region in (b) is indicative of (greater) aldol chemistry having taken place.

With the aid of the pure shift NMR techniques described already and of conventional multidimensional NMR we were able to establish the presence of comparable amounts of 2-aminooxazole **5**, *rac*-**15** and *rac*-**16**. The diastereoisomeric nature of *rac*-**15** and *rac*-**16** is supported by the pure shift DOSY data acquired using the pulse sequence shown in Figure 3 b. Figure 6 shows that the two species diffuse at the same rate, suggesting that they are of similar size. The chemistry of this mixture seems to have stalled at various intermediates and by-products in the pathway towards **5** (Scheme 3). This is reflected in the signal distribution of the pure shift DOSY spectrum of Figure 6, in which most of the species appear within a relatively narrow band of diffusion (and hence hydrodynamic radius). As mentioned previously, Cockerill et al. have shown that specific base catalysis improves the reaction yield for **5** to 40 % (Scheme 2).[30] General acid-base catalysis by phosphate is also efficient at pH 7, and results in a clean formation of **5** in>80 % yield.[23] Both types of catalysis presumably facilitate the 5-*exo*-dig cyclisation of the hydroxyl group onto the nitrile carbon of **10** to give **11**, as well as the C–H deprotonation of **17** leading to **3** (Schemes 2 and 3).[23] During the course of the reaction the pD rises to (and levels off at) pD 13. Despite this rise in pD, signals corresponding to potential aldol products were not identifiable, and it is assumed that if such chemistry had taken place, it was at low level. Further sequestration of cyanamide **7** by addition to *N*-cyano imine **18** to give **19**, and the oligomerisation of **7** in aqueous solution (see below), would result in the temporary excess of **6** required for the formation of *rac*-**15** and *rac*-**16**. 2-aminooxazole **5** is known to be a good C nucleophile at neutral pH, and reacts cleanly with various aldehydes and imines.[27, 45–47] For example, when isolated **5** is reacted with **6** in unbuffered water at neutral pH, the reaction proceeds in approximately 90 % yield, with a small diastereoselectivity for the kinetically-preferred *rac*-**15** over *rac*-**16** in a 46:43 ratio (Scheme 4).[27] However, the reaction mixture analysed in this pure shift DOSY study shows that *rac*-**16** predominated over *rac*-**15** (*rac*-**15**/*rac*-**16**, 1:1.7). If **6** and **7** are mixed in a 2:1 ratio, a greater formation of *rac*-**15** and *rac*-**16** is observed, with a slight relative increase in the diastereoselectivity for *rac*-**16** (*rac*-**15**/*rac*-**16**, 1:1.9), but now complexity between *δ*=3.4 and 3.8 ppm suggests that aldol chemistry had taken place due to the excess of **6** (with respect to **7**) in the reaction (Figure 7).

**Scheme 5:**
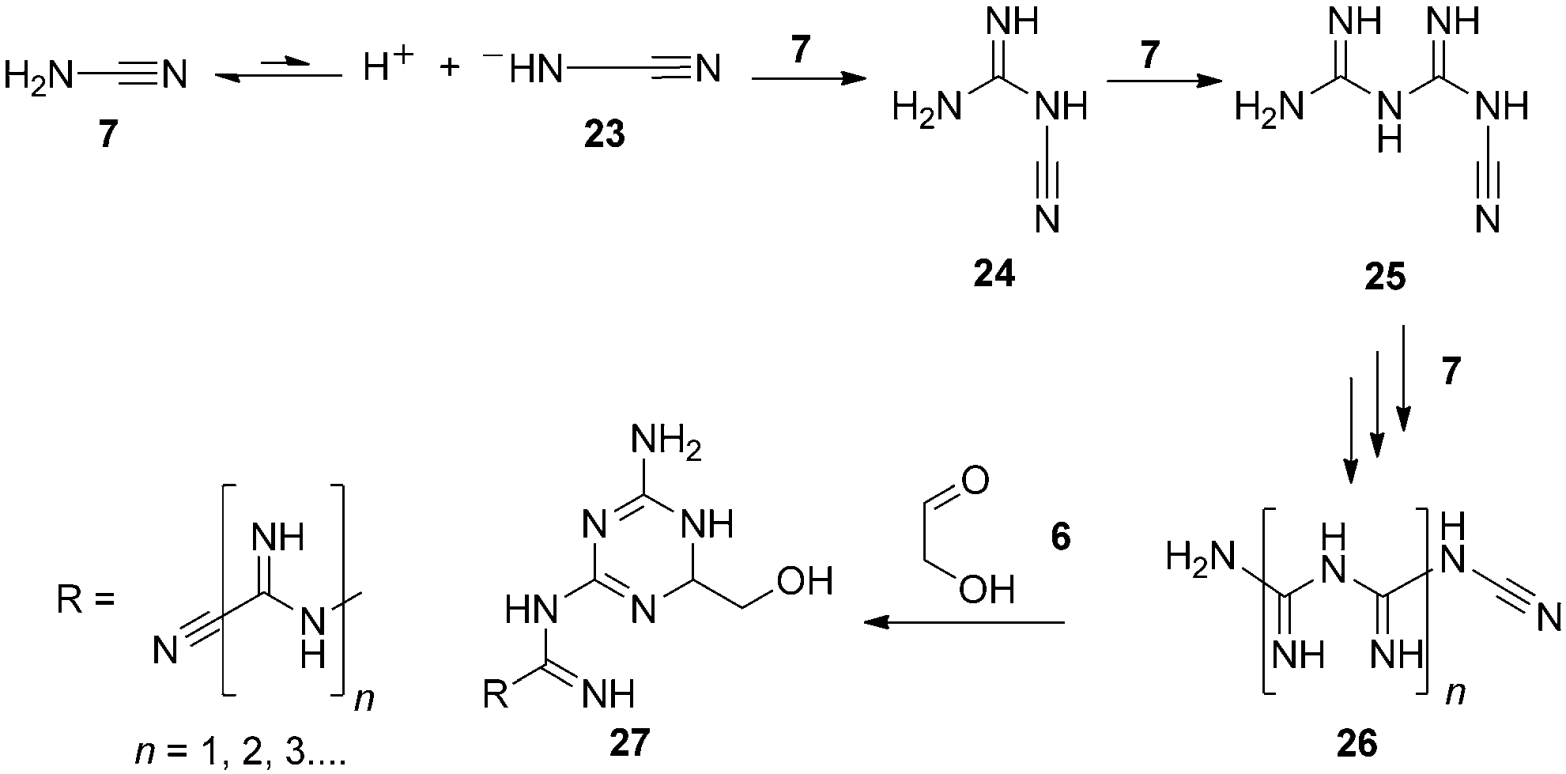
The oligomerisation of **7** and condensation with **6** to give cyclic aminal structures related to **27**.

Cyanamide **7** (p*K*_a_=10.3 in H_2_O)[48] is known to dimerise in aqueous solution.[49] The rise in pH would allow access to the conjugate base **23**, which could undergo nucleophilic attack onto **1** to give cyanoguanidine **24** (Scheme 5). Cyanoguanidine **24** is only partially soluble in water, and the observation of a small amount of white precipitate was indicative of this process. It is possible that further reaction of **24** with **7** could give rise to the formation of higher oligomeric material, with which **6** may have reacted to give cyclic aminal structures related to **27**, and two such species (**27 a** and **27 b**) were observed; similar aminal species have been observed in previous work from the addition of cyanamide **7** to sugar phosphates.[50] The nature of the R group of **27** cannot be determined in this study because of the shortage of NMR-observable atoms in the pendent side-chain (Scheme 5). Mass spectrometric analysis (ESI and APCI) of the reaction mixture did not yield any useful information. Nevertheless, the formation of such structures would explain why two structures related to **27** appear in Band 1 with lower diffusion coefficients (Figure 6).

## Conclusion

**Scheme 6:**
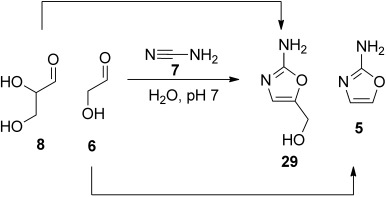
The formation of a mixture of **29** and **5** by treating glyceraldehyde **8** and glycolaldehyde **6** with cyanamide **7**.

**Scheme 7:**
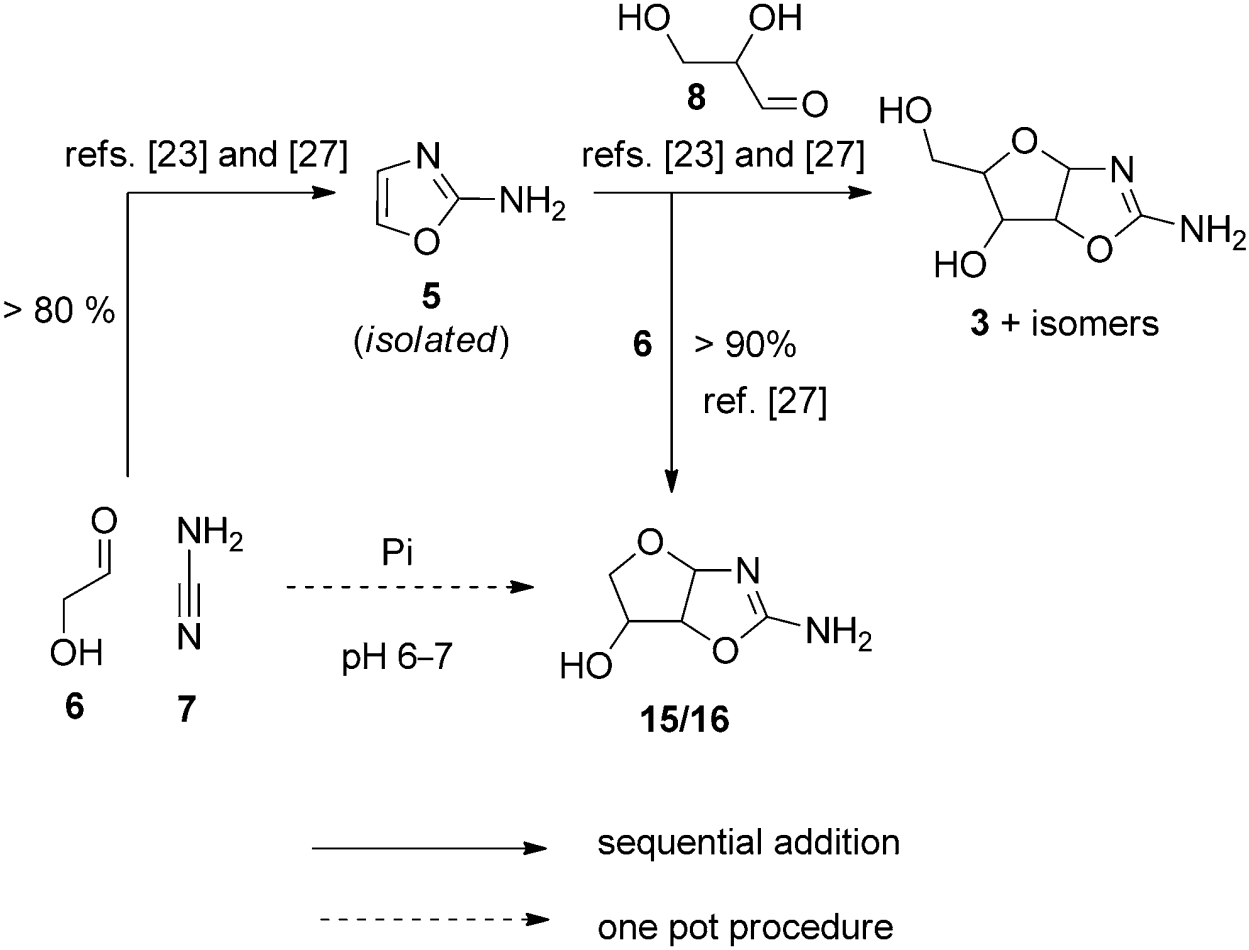
A potential one-pot synthesis of tetrose aminooxazolines **15** and **16**, without isolating 2-aminooxazole **5** (dashed arrows). Solid arrows are for known reactions that involve two separate-step operations, invoking a scenario in which **5** has to be produced in the absence of **8**.

During the course of the studies towards activated pyrimidine ribonucleotides **1** and **2**,[23] it became apparent that a potentially prebiotic assembly of pyrimidine threonucleotides from the same (or similar) chemical reactions that give **1** and **2** is also possible (Scheme 1). Previous studies in our laboratory had shown that there was no selectivity when carrying out a competitive reaction of cyanamide **7** with glycolaldehyde **6** and glyceraldehyde **8**. When treating **6** and **8** with cyanamide **7**, a 1:1 mixture of **5** and **29** was observed (Scheme 6, also see the Supporting Information). This observation currently necessitates a spatial and/or temporal separation requirement for the production of 2-aminooxazole **5** before reacting with glyceraldehyde **8** to form pentose aminooxazolines, such as **3**, en route to the activated pyrimidine ribonucleotides **1** and **2** (Scheme 1).[23] If an excess of glycolaldehyde **6** over cyanamide **7** is present, there is a possibility that the formation of tetrose aminooxazolines *rac*-**15** and *rac*-**16** would be favoured before 2-aminooxazole **5** has an opportunity to react with glyceraldehyde **8** (Scheme 7). Phosphate has been instrumental in controlling the reactivity in the synthesis of **1** and **2** (Scheme 1). Since its incorporation is required for nucleotide synthesis, the presence of phosphate at the very beginning of a reaction sequence is considered prebiotically more plausible, and is to be investigated in more detail. Any “pool” that produces pentose aminooxazolines from glyceraldehyde **8** and 2-aminooxazole **5** may also contain **15** and **16**.[27] Furthermore, a prebiotic origin of glyceraldehyde **8** still needs to be found for the synthesis of **1** and **2** to be fully accepted as a plausible route towards RNA. Since the prebiotic chemistry of a given bio-macromolecule should be assessed on the basis of generational criteria, preliminary observations here suggest that the abiogenesis of pyrimidine threonucleotides (and hence, TNA), in a similar manner to the production of **1** and **2**, is an intriguing possibility (Scheme 1). Ultimately, such speculation must be subjected to experimental assessment. Finally, the present work illustrates the need for new spectroscopic approaches to allow the analysis of increasingly complex mixtures without carrying out chromatographic separation of individual components. In this case it was made possible by the use of pure shift NMR, and it is anticipated that such techniques will have greater application in research involving mixed chemical systems in which it is undesirable to perturb the reaction composition.

## Experimental Section

**Pure shift NMR spectroscopic analysis**: The sample to be analysed by pure shift NMR was prepared as follows: glycolaldehyde **6** (54 mg, 0.89 mmol) and cyanamide **7** (37 mg, 0.89 mmol) were dissolved in D_2_O (1 mL) and stirred vigorously until all solids had dissolved. The pD at the start of the reaction was 7. The reaction was incubated at 40 °C, and the pD of the reaction gradually increased to 13 over a period of 48 h. The reaction was monitored by ^1^H NMR spectroscopy at intervals until signals corresponding to **6** had disappeared. The reaction mixture was then cooled to room temperature, spiked with *t*BuOH as an internal standard (*δ*_H_=1.25 ppm), and analysed by NMR spectroscopy. Pure shift and DOSY ^1^H NMR spectra were measured at ambient temperature on a Varian VNMRS 500 MHz spectrometer equipped with a 5 mm triple resonance probe and a *z* gradient coil giving a maximum gradient of 66 G cm^−1^. ^1^H-^13^C HSQC, ^1^H-^13^C HMBC and ^1^H-^1^H COSY spectra were acquired on a Bruker 500 MHz spectrometer equipped with a 5 mm triple resonance probe and a *z* gradient coil giving a maximum gradient strength of 53 G cm^−1^. The ^1^H-^13^C HSQC experiment of Figure 2 c was optimised for a 140 Hz ^1^H-^13^C coupling constant and data acquired using 4 transients and 512 increments. The DOSY spectrum of Figure 2 c was acquired using the Oneshot pulse sequence using 16 transients, one dummy scan and a 20 % imbalance factor.[35] The diffusion time D was 150 ms and the total diffusion-encoding gradient duration *δ* was 1.25 ms. Ten values of diffusion-encoding gradient were used, varying from approximately 10.5 to 41.8 G cm^−1^ in equal steps of gradient squared. The spectral width was 5387 Hz (32768 complex points). The pure shift data of Figures 5 and 6 all derive from a single experiment using the pulse sequence of Figure 3 b, slightly modified from that found previously reported in the literature,[32] with 32 transients per increment. The spectral width was 2003 Hz (16384 complex points). The Oneshot segment of the pulse sequence used the same parameters as for Figure 2 c; the Zangger–Sterk segment used a 37 ms (50 Hz) rsnob[36] selective pulse under a 0.23 G cm^−1^ gradient, with coherence transfer pathway selection pulses of width 1.5 ms with amplitude *G*_1_=31.31 G cm^−1^. Sixteen *t*_1_ increments were acquired with an *F*_1_ spectral width SW1 of 31.3 Hz; pure shift interferograms were assembled as described in the literature.[31] The assembled data were weighted with a Gaussian function of time constant half the maximum value of *t*_1_ before Fourier transformation. The pure shift ^1^H spectra of Figure 5 and 6 were, unusually, produced by co-adding the diffusion-weighted 1D spectra used to produce the corresponding DOSY spectra. This yields spectra that are slightly relaxation- and diffusion-weighted, which in the present case causes no significant problems, and avoids the need to perform a separate experiment to acquire a 1D pure shift spectrum. The signal-to-noise ratio of the 1D spectrum obtained in this way could have been slightly enhanced, at the expense of added complexity in data processing, by applying a suitable weighting as a function of diffusion-encoding gradient when co-adding the spectra. The 2D spectra were produced from the same data, by using the full FIDs instead of just the initial chunk needed to produce 1D pure shift spectra, and performing a standard 2D Fourier transform instead of 1D pure shift processing. The result is displayed in power mode to produce near-Lorentzian lines.

## Supplementary material

As a service to our authors and readers, this journal provides supporting information supplied by the authors. Such materials are peer reviewed and may be re-organized for online delivery, but are not copy-edited or typeset. Technical support issues arising from supporting information (other than missing files) should be addressed to the authors.
