# Supplementary material for: Detection of Potential TNA and RNA Nucleoside Precursors in a Prebiotic Mixture by Pure Shift Diffusion-Ordered NMR Spectroscopy
Source: Chemistry. 2013 Feb 1;19(14):4586–95. doi: 10.1002/chem.201202649 (PMC3814424; doi:10.1002/chem.201202649)
Supplement: Supplementary file 1 [file chem0019-4586-SD1.pdf]

CHEMISTRY
A EUROPEAN JOURNAL

Supporting Information

© Copyright Wiley-VCH Verlag GmbH & Co. KGaA, 69451 Weinheim, 2013

Detection of Potential TNA and RNA Nucleoside Precursors in a Prebiotic Mixture by Pure Shift Diffusion-Ordered NMR Spectroscopy

Saidul Islam,*[a, b] Juan A. Aguilar,*[b] Matthew W. Powner,[c] Mathias Nilsson,[b, d] Gareth A. Morris,[b] and John D. Sutherland[e]

chem_201202649_sm_miscellaneous_information.pdf

The supporting information contains-

1. Procedure for the preparation of sample analyzed by NMR.

2. Tabulated NMR data for compounds *rac*-**15**, *rac*-**16**, **5**, **14**, **27a** and **27b**.

3. Syntheses and characterization of compounds **5**, D-**15** and L-**16**.

4. Reactions of cyanamide in the presence of glyceraldehyde and glycolaldehyde.

4i. Formation of 2-amino-4-hydroxymethyloxazole **29** in aqueous solution.

4ii. Formation of 2-amino-4-hydroxymethyloxazole **29** and 2-aminooxazoline **5** in aqueous solution.

5. Standard DOSY spectrum of the sample analyzed in this manuscript.

6. Colour reproduction of Figures 5 and 6

1. Procedure for the preparation of sample analyzed by NMR

The sample analyzed by using pure shift NMR was prepared as follows. Glycolaldehyde **6** (54 mg, 0.89 mmol) and cyanamide **7** (37 mg, 0.89 mmol) were dissolved in D_2O (1 mL) and stirred vigorously until all solids had dissolved. The pD at the start of the reaction was 7. The reaction was incubated at 40 °C, and the pD of the reaction gradually increased over 48 h to 13. The reaction was monitored at various intervals until signals corresponding to **6** had disappeared by ^{1}H-NMR spectroscopy. The reaction was then cooled to room temperature and spiked with *t*-BuOH as an internal standard (δ_H = 1.25 ppm). The mixture was analyzed by NMR spectroscopy. Pure shift and DOSY ^{1}H NMR spectra were measured at ambient temperature on a Varian VNMRS 500 MHz spectrometer. ^{1}H-^{13}C HSQC, ^{1}H-^{13}C HMBC and ^{1}H-^{1}H COSY spectra were acquired on a Bruker 500 MHz spectrometer.

2. Tabulated NMR data for compounds *rac*-15, *rac*-16, 5, 14, 27a and 27b

NMR data obtained from the analysis of the mixture produced from the reaction of glycolaldehyde **6** and cyanamide **7**, described in section 1 of this supporting information, is presented in tabulated form. Further support for the assignment of *rac*-**15**, *rac*-**16** and **5** is presented in section 3, where their syntheses and characterization are described.

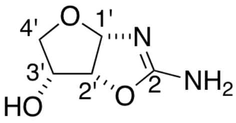

rac-**15**

Position	^{1}H	^{13}C	COSY	HMBC
1′	5.61 (*d*, *J* = 4.98)	98.16	2′	2
2′	4.75 (*t*, *J* = 5.24)	86.43	1′, 3′	-
3′	4.19 (*ddd*, *J* = 5.58, 6.67, 10.18)*	70.24	2′, 4′, 4′′	-
4′	3.82 (*dd*, *J* = 6.61, 8.96)	65.16	3′, 4′′	-
4′′	3.12 (*t*, *J* = 9.47)	65.16	3′, 4′	-
2	-	165.83	-	-

* *Coupling constants and multiplicity extrapolated from 2D ^{1}H pure shift DOSY data*

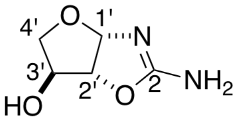

rac-**16**

Position	^{1}H	^{13}C	COSY	HMBC
1′	5.77 (*d*, *J* = 5.05)	97.75	2′	-
2′	4.70 (*d*, *J* = 5.40)	86.42	1′	2
3′	4.24 (*d*, *J* = 2.55)	73.34	4′, 4′′	
4′	3.82 (*dd*, *J* = 0.80, 10.93)	69.54	3′, 4′′	
4′′	3.12 (*dd*, *J* = 2.70, 10.94)	69.54	3′, 4′	
2	-	165.00	-	-

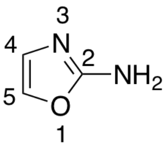

5

Position	^{1}H	^{13}C	COSY	HMBC
5	7.13 (*d*, *J* = 1.08)	133.03	5	-
4	6.60 (*d*, *J* = 0.95)	124.91	4	-
2	-	161.54	-	-

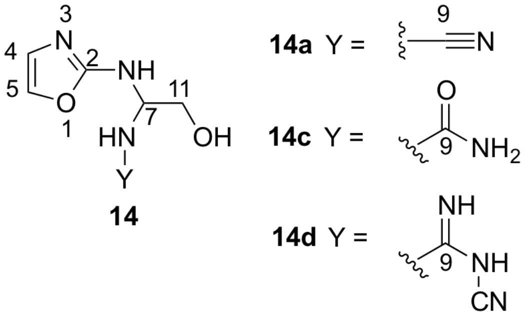

Position	^{1}H	^{13}C	COSY	HMBC
5	7.19 (*d*, *J* = 1.04)	133.92	4	2, 4
4	6.65 (*d*, *J* = 1.05)	124.91	5	2, 5
2	-	160.04	-	-
7	5.43 (*dd*, *J* = 4.42, 8.07)	72.56	11, 11′	9, 11, 11′
9	-	164.63	-	-
11	4.39 (*dd*, *J* = 8.04, 9.37)	72.66	7, 11	-
11′	4.09 (*dd*, *J* = 4.38, 9.42)	72.66	7, 11′	-

Comment: The general structure of this compound has been assigned as **14**. The NMR data of the aminooxazole moiety is distinctly similiar to the NMR data for **5**. The nature of the potential pendant groups **Y** is given as **14a**, **14c** and **14d** by consideration of the potential chemistries that may have taken place within the reaction (Scheme 3). The ^{13}C-NMR chemical shift for C9 suggests that the structures **14c** and **14d** are more likely than **14a**. Unambiguous characterization of this compound was not possible in the present study.

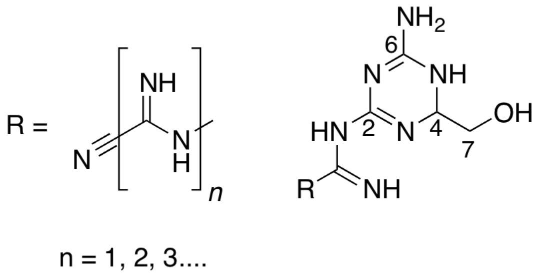

27a

Position	^{1}H	^{13}C	COSY	HMBC
4	5.38 (*dd*, *J* = 4.59, 8.35)	69.81	7, 7′	159.99
7	4.31 (*dd*, *J* = 7.87, 9.36)	73.07	4, 7′	-
7′	4.01 (*dd*, *J* = 5.54, 9.43)	73.07	4, 7	-

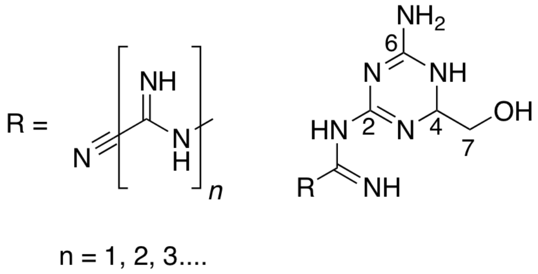

27b

Position	^{1}H	^{13}C	COSY	HMBC
4	5.32 (*dd*, *J* = 4.05, 8.02)	72.00	7, 7′	162.00, 164.62
7	4.34 (*dd*, *J* = 7.96, 9.38)	73.10	4, 7′	164.62
7′	4.01 (*dd*, *J* = 7.96, 9.38)	73.10	4, 7	164.62

3. Synthesis and characterization of compounds 5, D-15 and L-16

The syntheses and characterization of **5**, D-**15 and** L-**16** are presented here to lend further support to the structural assignments of these three substrates obtained from the NMR analysis of the mixture obtained from the mixing of glycolaldehyde **6** and cyanamide **7** (as described in section 1, and structural assignments given in section 2).

2-aminooxazole ***5***

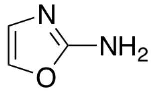

To a solution of glycolaldehyde **6** (0.6 g, 10.0 mmol) in water (5 mL) was added cyanamide **7** (420 mg, 10.0 mmol), and the pH of the solution was adjusted to pH 10 with 1M NaOH. The solution was stirred at 60 °C for 3 h, cooled and extracted into EtOAc (3 x 50 mL). The combined organic layers were dried over $MgSO_4$, and concentrated *in vacuo*, and recrystallised from heptane:EtoAc to yield 2-aminooxazole **5** (0.4 g, 50%) as long thin clear needles. R_f 0.73 (MeOH:EtOAc 1:4). M.p. 93-95°C. ^{1}H-NMR (400 MHz, $CDCl_3$) δ 7.12 (d, J = 1.0 Hz, 1H, **H**-(CO)); 6.73 (d, J = 1.0 Hz, 1H, **H**-(CN)); 5.44 (br. s, 2H, -N**H**$_2$). ^{13}C-NMR (100 MHz, $CDCl_3$) δ 161.0 (**C**-NH_2); 132.3 (H-(**C**O)); 126.4 (H-(**C**N)). ESI-MS (pos. *m/z*): 85 (100%, $[M+H]^+$). EI-HRMS (neg. *m/z*): $[M]^-$ calculated for $C_3H_4ON_2$, 84.0318; found 84.0321.

D-*Erythrose aminooxazoline* ***15***

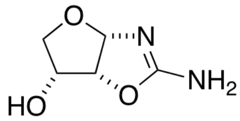

D-Erythrose (150 mg, 1.25 mmol) was dissolved in water (12.5 mL) to which cyanamide **7** (105 mg, 2.50 mmol) was added. The pH of the solution was adjusted to 7 using dilute NaOH and heated at 60°C for 9h followed by cooling and lyophilisation to yield 270 mg as a pale yellow syrup. Analysis by ^{1}H NMR showed conversion of starting material to D-**15**, alongside other minor unidentifiable species. ^{1}H NMR (500 MHz, D_2O) δ 5.70 (d, J = 4.7 Hz, 1H, **H**-(C1′)); 4.82 (t, J = 5.4 Hz, 1H, **H**-(C2′)); 4.21 - 4.24 (m, 1H, **H**-(C3′)); 3.88 (dd, J = 9.0, 6.7 Hz, 1H, **H**-(C4′)); 3.18 (t, J = 9.5 Hz, 1H, **H**-(C4″)). ^{13}C NMR (100 MHz, D_2O) δ 171.3 (C=N); 98.3 (C1′); 81.2 (C2′); 70.5 (C3′); 65.42 (C4′). ESI-MS (pos. *m/z*): 145 (100%, $[M+H]^+$). ESI-HRMS (pos. *m/z*): calculated $[M+H]^+$ for $C_5H_9O_3N_2$ 145.0608; found 145.0612.

L-*Threose aminooxazoline* ***16***

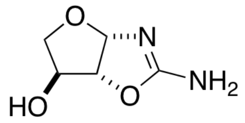

L-Threose (49 mg, 0.41 mmol) was dissolved in water (4 mL) to which cyanamide (34 mg, 0.82 mmol) was added. The pH of the solution was adjusted to 7 using dilute NaOH and heated at 60°C for 3h followed by cooling and lyophilisation to yield 70 mg as an off-white solid. Analysis by ^{1}H NMR showed complete conversion of starting material to and other minor unidentifiable species. An analytical sample (10 mg) was obtained by recrystallisation from hot water. R_f 0.03 (MeOH:EtOAc 1:9). M.p. 154-155 °C. ^{1}H NMR (500 MHz, D_2O) δ 5.83 (d, J = 5.0 Hz, 1H, **H**-(C1′)); 4.77 (d, J = 5.0 Hz, 1H, **H**-(C2′)); 4.31 (d, J = 2.5 Hz, 1H, **H**-(C3′)); 3.88 (d, J = 11.0 Hz, 1H, **H**-(C4′)); 3.55 (dd, J = 10.9, 2.7 Hz, 1H, **H**-(C4″)). ^{13}C NMR (125 MHz, D_2O) δ 165.1 (C=N); 97.8 (C1′); 86.4 (C2′); 73.3 (C3′); 70.0 (C4′). ESI-MS (pos. *m/z*): 145 (100%, $[M+H]^+$). ESI-HRMS (pos. *m/z*): $[M+H]^+$ calculated for $C_5H_9O_3N_2$ 145.0608; found 145.0607.

4. Reactions of cyanamide 7 in the presence of glyceraldehyde 8 and glycolaldehyde 6

No selectivity was observed when cyanamide **7** was treated with glycolaldehyde **6** and *rac*-glyceraldehyde **8**. Upon treatment of a mixture of these two α-hydroxyaldehydes with **7**, a mixture of products **5** and **29** was observed (Scheme S1). This implies the requirement for spatial and/or temporal separation of the production of **6** and **8** for the formation of pentose aminooxazolines *en route* to the synthesis of activated pyrimidine ribonucleotides.[S1] **29** was formed more slowly than **5**, taking 18 days in aqueous solution *via* the intermediacy of diastereoisomeric adducts **30** and **31** and the imino-tautomer **32** as observed by ^{1}H NMR spectroscopy. **5** can be separated from this mixture (*vide infra* section 3ii) and is known to sublime,[S2] but further investigation towards separation and the subsequent reaction with **8** were not pursued in this study.

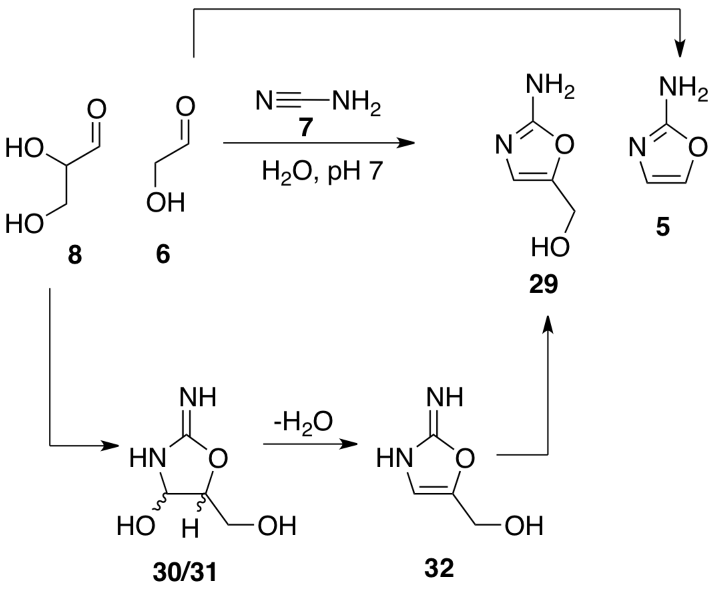

Scheme S1. Formation of mixed 2-aminooxazole products **5** and **29** through treatment of a mixture of α-hydroxyaldehydes **6** and **8** with cyanamide **7**.

4i. Formation of 2-amino-4-hydroxymethyloxazole 29 **in aqueous solution**

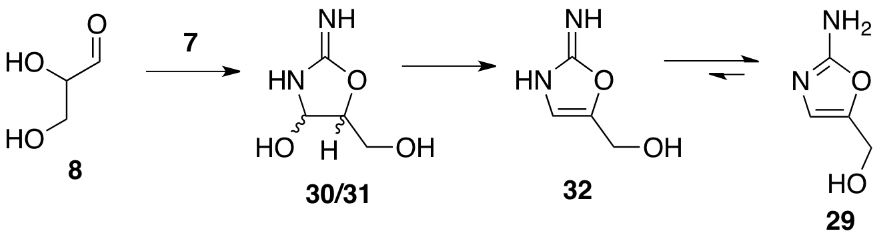

rac-Glyceraldehyde **8** (90 mg, 1.0 mmol) was dissolved in D_2O (1 mL) at pD 7.0. Cyanamide **7** (42 mg, 1.0 mmol) was added and the solution was incubated at 22 °C and analysed by ^1H NMR analysis. Two diastereomeric adducts **30** and **31** initially formed at a ratio of 5.5:4.5, then 2-imino-4-hydromethyloxazole **32** and 2-amino-4-hydroxymethyloxazole **29** slowly formed. Complete conversion to **29** was observed after 18 days.

^1H NMR (500 MHz, D_2O) δ_H Major isomer **30**: 5.08 ppm (d, J = 2.7 Hz, 1H, H-(C5)); 4.26 (ddd, J = 5.5, 3.3, 3.0 Hz, 1H, H-(C4)); 3.66 (ABX, J = 12.8, 3.5 Hz, 1H, CH_2); 3.51 (ABX, J = 12.8, 5.4 Hz, 1H, CH_2).

^{1}H NMR (500 MHz, D_2O) δ_H Minor isomer **31**: 5.16 ppm (d, *J* = 4.8 Hz, 1H, H-(C5)); 4.42 (td, *J* = 5.0, 3.5 Hz, 1H, H-(C4)); 3.74 (dd, *J* = 12.5, 3.2 Hz, 1H, CH_2); 3.59 (dd, *J* = 12.5, 5.2 Hz, 1H, CH_2).

^{1}H NMR (500 MHz, D_2O) δ_H 2-Imino-4-hydromethyloxazole **32**: 6.59 ppm (s, 1H, H-(C5)); 4.35 (s, 2H, CH_2).

^{1}H NMR (500 MHz, D_2O) δ_H 2-Amino-4-hydroxymethyloxazole **29**: 6.54 ppm (s, 1H, H-(C5)); 4.33 (s, 2H, CH_2).

4ii. Formation of 2-amino-4-hydroxymethyloxazole 29 **and 2-aminooxazoline** 5 **in aqueous solution**

rac-Glyceraldehyde **8** (45 mg, 0.5 mmol) and glycolaldehyde **6** (30 mg, 0.5 mmol) were dissolved in D_2O (1 mL) at pD 7.0. Cyanamide **7** (42 mg, 1.0 mmol) was added and the solution was incubated at 60 °C for 5 h. The formation of **29** and **5** were observed in a 1:1 mixture by spectral comparison. The sample was then dried *in vacuo* and heated at 60 °C for 16 h. The resultant crystalline sublimate product was collected to give 3 mg (7%) and dissolved in D_2O (0.75 mL) for NMR analysis. NMR spectral data for the sublimate corresponded to 2-aminooxazole **5**.

^{1}H NMR (500 MHz, D_2O) δ_H 2-Amino-4-hydroxymethyloxazole **29**: 6.54 ppm (s, 1H, H-(C5)); 4.33 (s, 2H, CH_2).

^{1}H NMR (500 MHz, D_2O) δ_H 2-Aminooxazole **5**: 7.18 ppm (d, *J* = 1.0 Hz, 1H, H-(C4)); 6.65 (d, *J* = 1.0 Hz, 1H, H-(C5)).

5. Standard DOSY spectrum of the sample analyzed in the paper

The Doneshot pulse sequence was used to acquire the following standard DOSY spectrum.

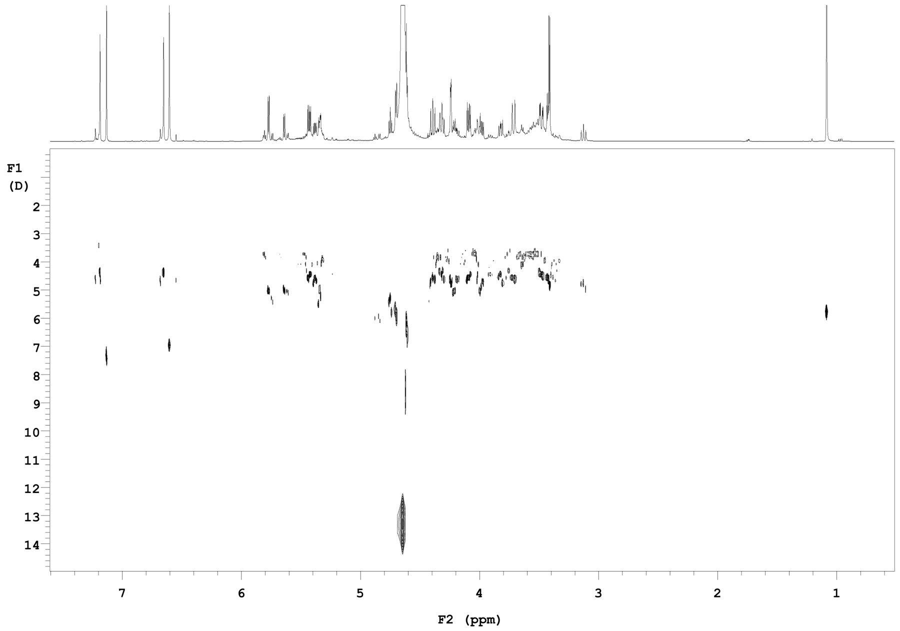

6. Colour reproduction of Figures 5 and 6

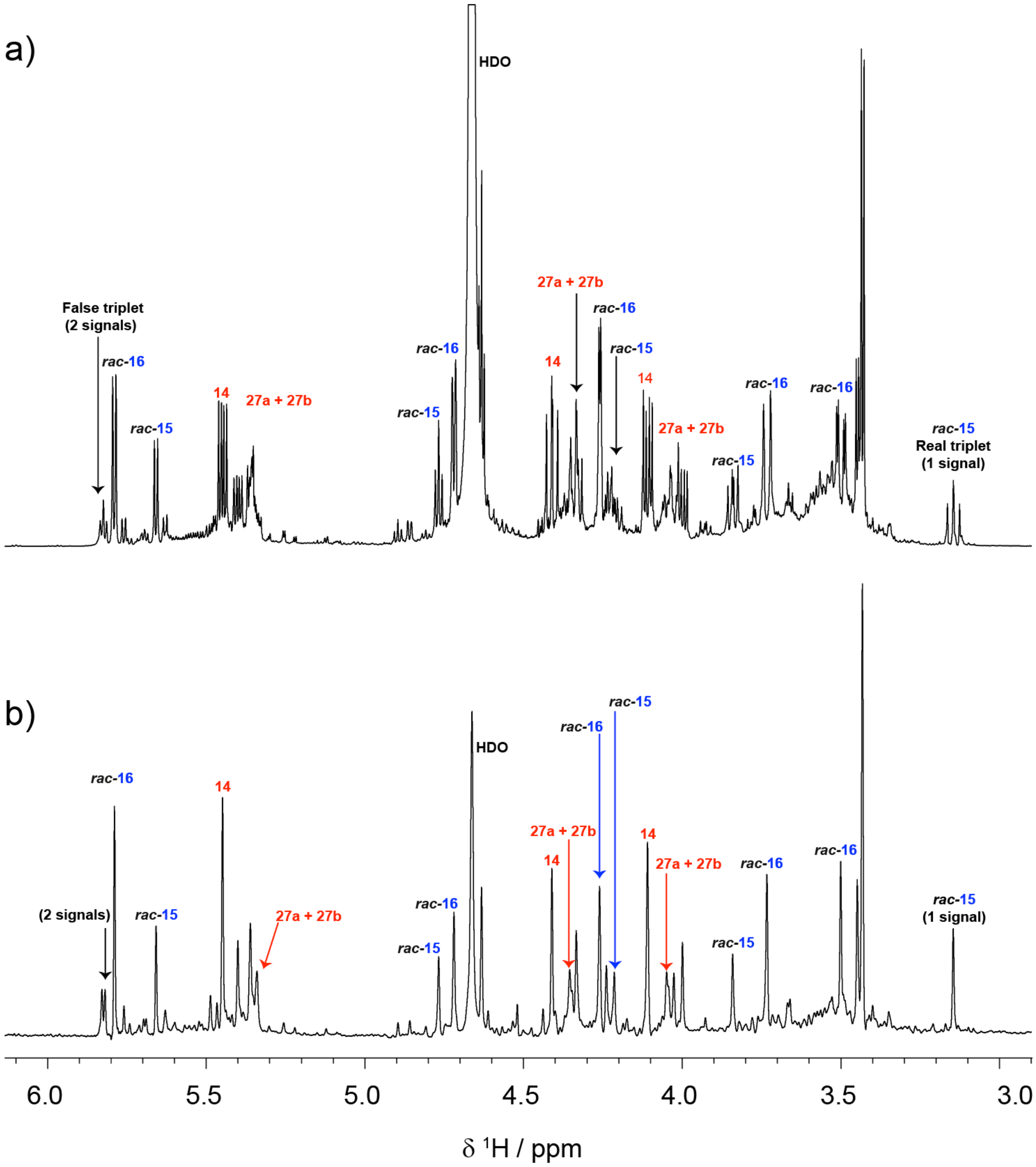

Figure 5. ^{1}H (a) and pure shift ^{1}H spectra (b) of the highly overlapped region, which include signals of the potential TNA precursors tetrose aminooxazolines *rac*-**15** and *rac*-**16** amongst various other adducts.

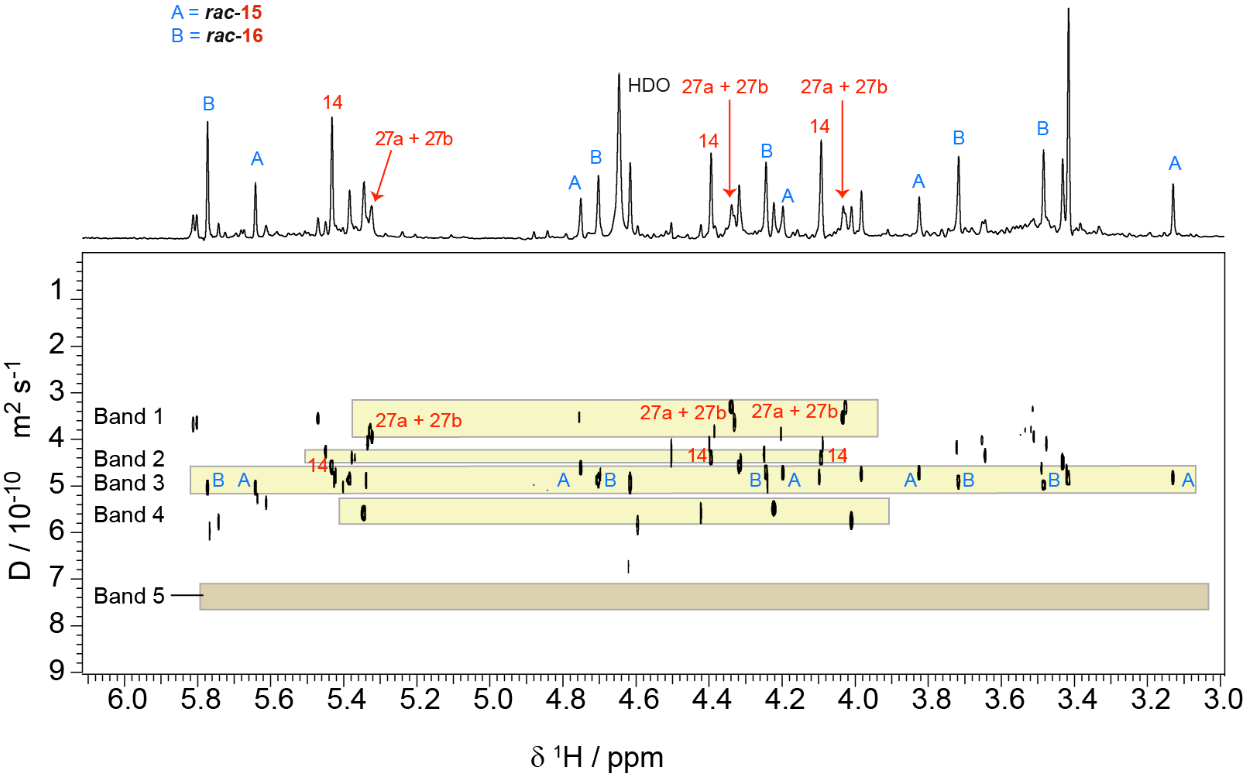

Figure 6. ^{1}H pure shift Oneshot DOSY spectrum obtained with the pulse sequence of Figure 3b; shaded rectangles are used to highlight particular diffusion ranges. The corresponding spectrum for the aromatic region (6.5-7.5 ppm) is shown in Figure 2. The lack of signals in Band 5 supports the assignment of **5** in Figure 2, while the aliphatic signals of **14** are seen in Band 2. Diastereoisomeric tetrose aminooxazolines *rac*-**15** and *rac*-**16** share the same diffusion band as expected. The structures and assignments shown are based on the combined evidence of Figure 2, Figure 4, Figure 5, and of the conventional 2D-NMR experiments

References

(S1) M. W. Powner; B. Gerland; J. D. Sutherland *Nature* **2009**, *459*, 239.

(S2) C. Anastasi; M. A. Crowe; M. W. Powner; J. D. Sutherland *Angew. Chem.*, **2006**, 118, 6322; *Angew. Chem. Int. Ed.* **2006**, *45*, 6176.
